# An effective therapeutic regime for treatment of glioma using oncolytic vaccinia virus expressing IL-21 in combination with immune checkpoint inhibition

**DOI:** 10.1016/j.omto.2022.05.008

**Published:** 2022-06-06

**Authors:** Yijie Sun, Zhe Zhang, Chenglin Zhang, Na Zhang, Pengju Wang, Yongchao Chu, Louisa S. Chard Dunmall, Nicholas R. Lemoine, Yaohe Wang

**Affiliations:** 1 National Centre for International Research in Cell and Gene Therapy, School of Basic Medical Sciences, Academy of Medical Sciences, Zhengzhou University, Zhengzhou 450052, China; 2 Centre for Cancer Biomarkers & Biotherapeutics, Barts Cancer Institute, Queen Mary University of London, London EC1M 6BQ, UK

**Keywords:** oncolytic vaccinia virus, glioblastoma, α-PD1, intravenous injection, anti-tumor immunity, IL-21

## Abstract

Glioblastoma (GBM) is the most common primary malignant tumor in the brain, accounting for 51.4% of all primary brain tumors. GBM has a highly immunosuppressive tumor microenvironment (TME) and, as such, responses to immunotherapeutic strategies are poor. Vaccinia virus (VV) is an oncolytic virus that has shown tremendous therapeutic effect in various tumor types. In addition to its directly lytic effect on tumor cells, it has an ability to enhance immune cell infiltration into the TME allowing for improved immune control over the tumor. Here, we used a new generation of VV expressing the therapeutic payload interleukin-21 to treat murine GL261 glioma models. After both intratumoral and intravenous delivery, virus treatment induced remodeling of the TME to promote a robust anti-tumor immune response that resulted in control over tumor growth and long-term survival in both subcutaneous and orthotopic mouse models. Treatment efficacy was significantly improved in combination with systemic α-PD1 therapy, which is ineffective as a standalone treatment but synergizes with oncolytic VV to enhance therapeutic outcomes. Importantly, this study also revealed the upregulation of stem cell memory T cell populations after the virus treatment that exert strong and durable anti-tumor activity.

## Graphical abstract

**Figure:**
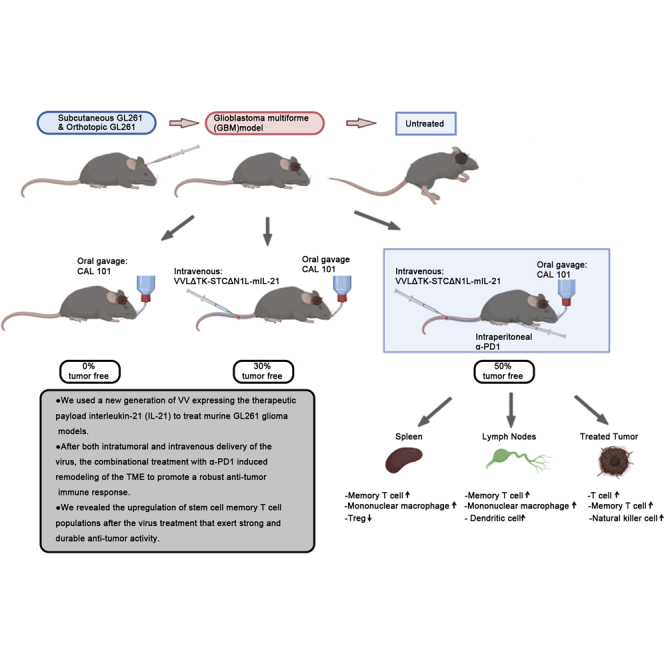

## Teaser

Oncolytic vaccinia virus armed with interleukin-21 is an effective treatment in subcutaneous and orthotopic models of glioma and synergizes with α-PD1 to promote tumor clearance. Treatment results in induction of potent adaptive immune responses, including stem cell memory T cell responses that exert strong and durable anti-tumor activity.

## Introduction

Glioblastoma multiforme (GBM) is an extremely malignant tumor of the central nervous system characterized by high heterogeneity, invasiveness, and recurrence. GBM is the most common primary malignant tumor in the skull, accounting for about 51.4% of all primary brain tumors.^1^ At present, conventional treatment involves maximal tumor resection combined with temozolomide chemotherapy and radiotherapy. However, 90% of patients still die within 2 years of diagnosis.^2^^,^^3^ GBM is considered an immunologically inert or “cold” tumor as tumor-infiltrating lymphocytes are largely excluded from the tumor microenvironment (TME) and those remaining tend toward exhausted phenotypes.^4^^,^^5^ As such, GBM is resistant to current immunotherapeutic interventions, such as immune checkpoint inhibition (ICI), aimed at potentiating anti-tumor immune responses. New therapeutics that can reverse the immunosuppressive environment of GBM, rendering GBM susceptible to immune system attack, are therefore urgently required.

Oncolytic viral therapy (OVT) is an extensively studied new immuno-therapeutic approach designed to selectively destroy cancer cells through virus replicating specifically within tumor cells.^6^ One of the most important aspects of OVT is its ability to induce immunogenic cell death (ICD) of tumor cells and harness an immune response at the site of the tumor. The activation of pathogen-associated molecular pattern signals and damage-associated molecular pattern signal pathways consequent to viral infection can overcome the immunosuppressive nature of the TME,^7^ initiating an efficient tumor-specific immune response and forming long-term tumor-specific immune memory.^8^, ^9^, ^10^ In addition, OVTs can carry various therapeutic genes to the tumor site, where they are produced and expressed locally at high levels. OVTs have demonstrated strong potential for the treatment of brain malignancies as they do not antagonize traditional treatments and are associated with low levels of toxicity. Indeed Japan has recently approved an oncolytic herpesvirus (HSV), G47Δ, for treatment of adult patients with malignant glioma, having demonstrated impressive efficacy after stereotactic injection during clinical trials.^11^

Vaccinia virus (VV) has many advantageous features compared with other OVTs, including but not limited to: (1) its rapid life cycle, which only takes 6 h to produce mature progeny virus;^12^ (2) multiple infectious forms that ensures rapid and effective transmission of the virus;^13^ (3) lack of requirement for specific cell surface receptors;^14^^,^^15^ (4) its large viral genome that can accept foreign DNA inserts of up to 25 kb;^16^ (5) its ability to replicate in hypoxic environments;^17^ and (6) its safety record through a rich history of clinical application.^18^ In addition, VV induces ICD pathways to activate immune responses and infection results in vascular collapse in the TME.^19^^,^^20^

The oncolytic VV Lister strain used in this study has previously undergone rational gene editing to enhance its oncolytic effect, while reducing side effects.^21^ Thymidine kinase and N1L gene deletions ensure tumor selectivity^22^ and the deletion of N1L provides an additional advantage of significant improvement in induction of anti-tumor immune responses via multiple mechanisms, including improved T cell infiltration to tumors, improved activation of T cells, and increased circulating natural killer (NK) cells.^23^ In addition, the virus expresses a second, mutant copy of the viral B5R gene that we have previously shown to be essential for extracellular enveloped virus (EEV) production and therefore long-range spread of the virus. Indeed, after intravenous (i.v.) delivery of the virus with this modification, significantly more viral genome copies were detected in subcutaneous tumors and survival was improved in Syrian hamster models of disseminated pancreatic cancer.^21^ We have also previously described an effective systemic delivery platform for VV via transient inhibition of PI3Kδ to prevent macrophage uptake of systemically delivered VV. The development of an i.v. delivery platform of VV is crucial for glioma treatment because of its intracranial location, which poses a safety concern for i.t. injection of therapeutics.^21^^,^^24^

Interleukin-21 (IL-21) is an extremely attractive cytokine in the context of anti-tumor immunotherapy as it can safely and effectively enhance immunity to tumors through multiple mechanisms. IL-21 is an effective inducer of T cell activation *in vivo*, enhances the antigen affinity of specific CD8+ T cells,^25^^,^^26^ inhibits the development of regulatory T cells (Treg),^27^ induces the maturation of NK and NKT cells, induces activation and cytolytic potential of NK and NKT cells,^28^^,^^29^ promotes the production of tumor-specific IgG B cells,^30^ and inhibits angiogenesis by reducing the expression of VEGFR1 and TIE1 in endothelial cells.^31^ Most importantly, even high-dose delivery of IL-21 has not resulted in development of toxic side effects often noted with other similarly pleiotropic cytokines.^30^ ICI are currently being investigated as a therapeutic option for glioma. Promising therapeutic activity has been noted in preclinical glioblastoma models, but the results of clinical trials in patients with recurrent glioblastoma are disappointing, likely due to the low mutational burden and poor T cell infiltration in GBM.^32^ OVs have previously been shown by us and others to synergize effectively with ICI.^21^^,^^33^, ^34^, ^35^ OVs can efficiently reprogram the TME, recruiting anti-tumor immune cells. However, tumor cells can shut down activation of T cells via rapid activation of the immune checkpoints. The addition of ICI therapy to the regime is therefore likely to be of additional therapeutic benefit by preventing the inactivation of newly recruited T cells.

Here, we demonstrate that a VV expressing IL-21, VVΔTK-STCΔN1L-mIL-21 is effective at evoking anti-tumor immunity and eliminating tumors after both i.t. and i.v. injection to glioma models. The anti-tumor efficacy was enhanced in combination with the ICI α-PD1. We demonstrated that treatment was able to effectively remodel the TME to promote anti-tumor immune responses and importantly found that it enhanced a population of memory T cells known as stem cell memory T cells (TSCMs) that have previously been shown to exert strong anti-tumor activity.^36^

## Results

### VVLΔTK-STCΔN1L-mIL-21 and VVLΔTK-STCΔN1L replicate in, are cytotoxic to, and express mIL-21 in murine GBM cell lines

**Figure 1:**
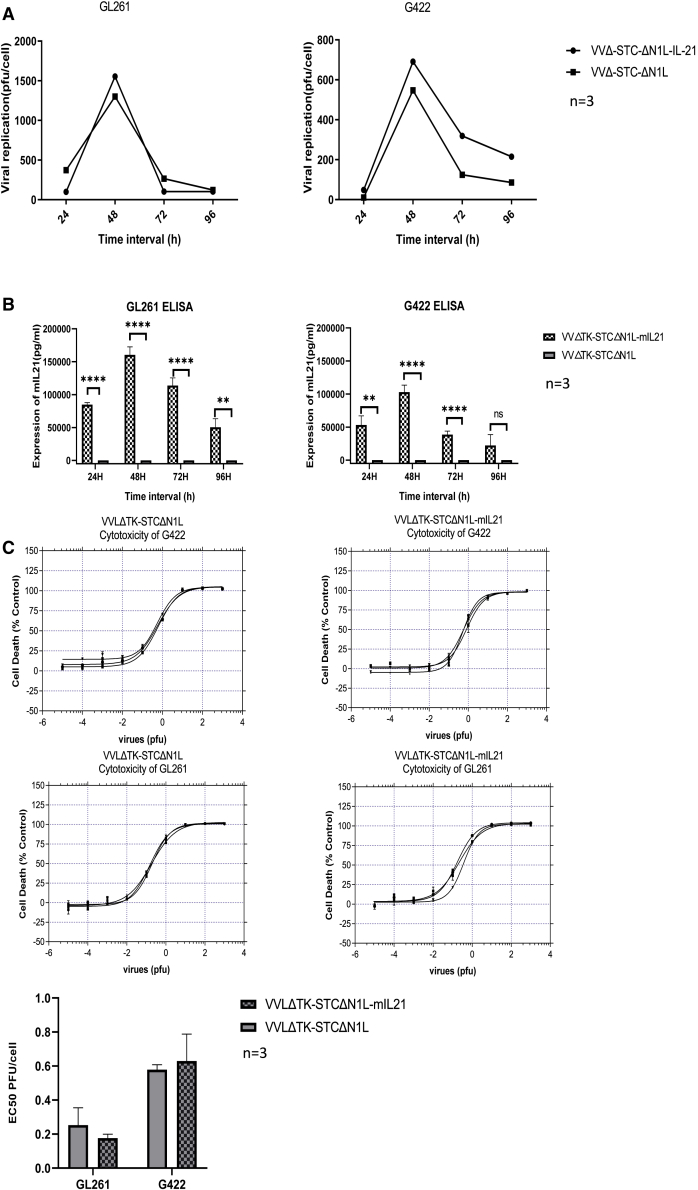
VVLΔTK-STCΔN1L and VVLΔTK-STCΔN1L-mIL-21 replicate in and are cytotoxic to glioma cell lines (A) GL261 and G422 were infected with VV and cells and supernatant harvested at 24–96 h post-infection (hpi). Virus was titered using TCID_50_ assay and the mean PFU/cell shown and significance tested using Student’s unpaired t test (n = 3/group). (B) Aliquots of supernatant from (A) were assessed for IL-21 expression using ELISA. Mean values at each time point are shown and significance tested using Student’s unpaired t test (n = 3/group). (C) Virus cytotoxicity to the cell lines was detected by the MTS assay 6 days following infection. The mean EC_50_ values of the two viruses is shown (n = 3/group) and significance tested using Student’s unpaired t test. ∗∗p < 0.01, ∗∗∗p < 0.001, ∗∗∗∗p < 0.0001.

To determine the ability of the viruses to replicate in murine GBM cell lines, murine GL261 and G422 cells were infected at a multiplicity of infection (MOI) of 1 PFU/cell for 24–96 h. Median tissue culture infective dose (TCID_50_) analysis of viral titers at each time point indicated that both the control and mIL-21 virus replicated effectively in each cell line, with replication peaking at 48 h post-infection (Figure 1A). Efficient mIL-21 expression was confirmed in cell supernatant at each time point using ELISA (Figure 1B).

To investigate cytotoxicity in the same cell lines, cell cytotoxicity (MTS) assays were performed. Both viruses demonstrated a low EC_50_ value (Figure 1C), indicating strong cytotoxicity against both cell lines. A comparison of the viruses demonstrated that there was no significant difference between the control and IL-21-containing virus, indicating that the addition of mIL-21 expression does not affect the cytotoxic effect affect the cytocytotoxic of the virus *in vitro* (Figure 1C).

### VVLΔTK-STCΔN1L-mIL-21 can effectively inhibit tumor growth and eliminate GL261 subcutaneous tumors in mice and enhance the therapeutic effect of α-PD1

**Figure 2:**
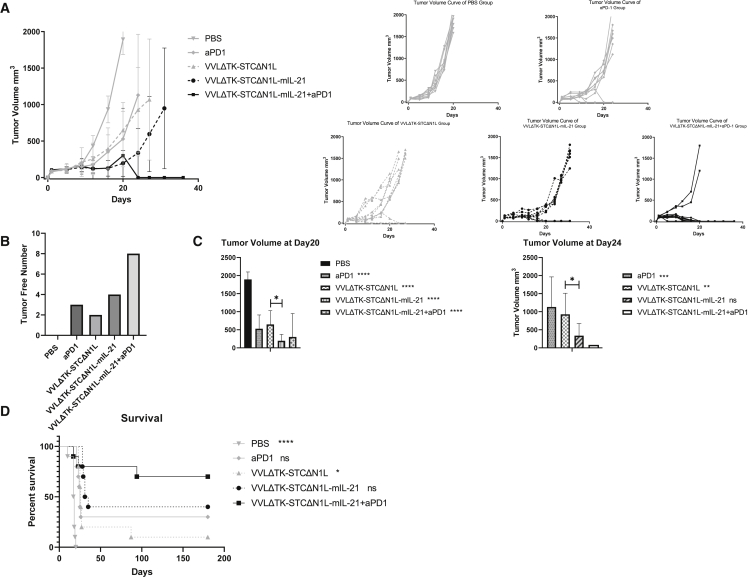
VVLΔTK-STCΔN1L combined with α-PD1 is an effective treatment for murine GL261 subcutaneous tumors (A) GL261 subcutaneous tumors were established in immunocompetent mice (n = 10/group) and treated six times with the indicated virus (± three injections of α-PD1). Tumor growth was measured twice weekly and the growth curve combining groups (left) and for each individual (right) (mean/group ± SD) are presented. One biological repeat was carried out. (B) The number of tumors completely cleared after treatment, without recurrence during the observation period is shown (n = 10/group). (C) Mean tumor volumes at day 20 (the time point that PBS group tumor volume reached 1,500 mm^3^) and 24 (the time point that tumor volume of mice in other control treatment groups reached 1,500 mm^3^) after the first treatment are shown. Significance difference in relation with PBS group (day 20) and the combination treatment group (day 24) tested using a one-way ANOVA with Bonferroni post-hoc testing are shown on the legends (n = 10/group). (D) Kaplan-Meier survival analysis of mice in each group. Log rank (Mantel-Cox) testing was used to determine the significance. All treated groups were significantly different from PBS group. The significance of the comparison of each group with VVLΔTK-STCΔN1L-mIL-21 + α-PD1 group is noted on the legend (n = 10/group). ∗p < 0.05, ∗∗ p < 0.01, ∗∗∗p < 0.001, ∗∗∗∗p < 0.0001.

Subcutaneous GL261 tumors were established in immunocompetent C57BL/6 mice, and animals were treated with six i.t. injections of VVLΔTK-STCΔN1L-mIL-21 or VVLΔTK-STCΔN1L (1 × 10^8^ PFU/injection) on days 1, 3, 5, 14, 16, and 18, a regime previously determined as effective for the robust treatment of pancreatic cancer.^21^ When used in combination, α-PD1 was administered intraperitoneally (i.p.) on days 2, 4, and 6. Both the control virus (VVLΔTK-STCΔN1L) and α-PD1 were able to delay tumor growth and cure 2/10 and 3/10 mice, respectively. The therapeutic virus (VVLΔTK-STCΔN1L-mIL-21) was significantly more effective than either of these treatments, leading to long-term survival in 4/10 mice treated. However, the combination of VVLΔTK-STCΔN1L-mIL21 with α-PD1 therapy demonstrated the most powerful therapeutic effect, with 80% cure and no recurrence in the 180 day observation period (Figures 2A–2D). These results demonstrate the efficacy of combining OVT with concurrent α-PD1 therapy to achieve a strong therapeutic effect.

### VVLΔTK-STCΔN1L-mIL-21 in combination with α-PD1 enhances anti-tumor immunity

We performed flow cytometric analysis on mouse splenocytes on days 7, 14, and 21 post-infection to determine the effect of treatment on T cell, dendritic cell (DC), and macrophage (Figure S1) populations.

**Figure 3:**
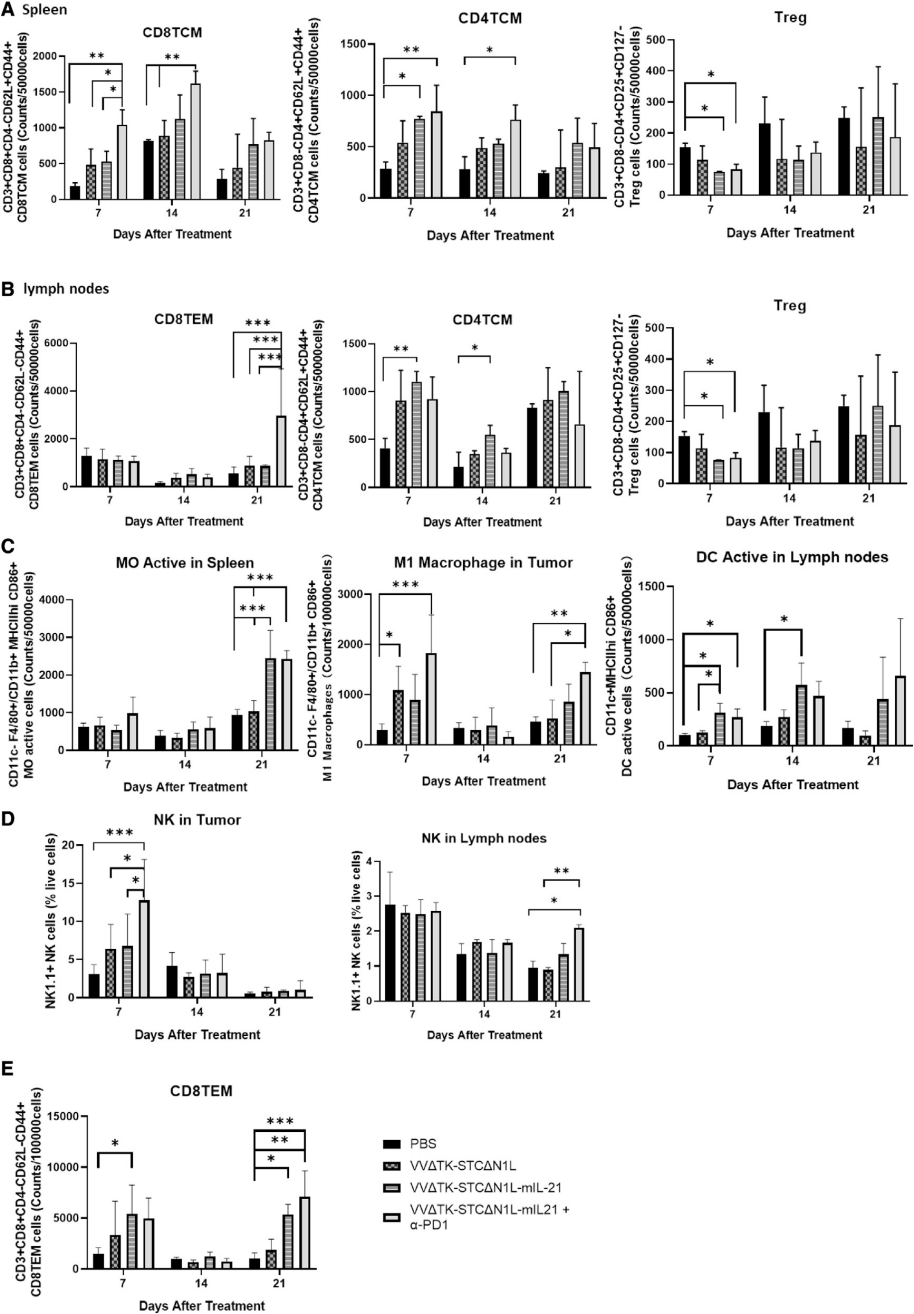
Immune cells are altered in mouse tissues after treatment with VVΔTK-STCΔN1L-mIL21+ α-PD1 (A) CD8+, CD4+, and Treg cells were quantified in the spleens at the indicated time points after treatment. (B) CD8+, CD4+, and Treg cells were quantified in the lymph nodes at the indicated time points after treatment. (C) The innate immune cells in the spleen, lymph nodes, and tumors of mice were examined at the indicated time points after treatment. (D) NK cells frequencies quantified in lymph nodes and tumor as a total of live cells. (E) CD8+ TEM cells quantified in tumor tissues (n = 3–4/group). Significance was analyzed for each panel using a two-way ANOVA. ∗p < 0.05, ∗∗p < 0.01, ∗∗∗p < 0.001.

Analysis of T cell populations demonstrated that splenic CD8+ TCM (central memory T cell) in each treatment group was significantly increased compared with the PBS group by day 7 after the first treatment. The increase in the VVΔTK-STCΔN1L-mIL21 + α-PD1 group was significantly higher compared with other groups (Figure 3A, left panel) and was sustained through to day 14 post-treatment. This group also resulted in a CD4+ TCM response (Figure 3A, middle panel) until day 14, and both VVΔTK-STCΔN1L-mIL21 and VVΔTK-STCΔN1L-mIL21 + α-PD1 groups were able to statistically reduce splenic regulatory T cell (Treg) populations 7 days post-treatment (Figure 3A, right panel). Significantly increased splenic CD4+ and CD8+ responses to viral and tumor antigens reduced by day 21, likely as viral infection resolved and tumor burden decreased. Similar analysis of the lymph node compartment revealed that both VVΔTK-STCΔN1L-mIL21 and VVΔTK-STCΔN1L-mIL21 + α-PD1 groups were able to statistically reduce lymph node Treg populations 7 days post-treatment (Figure 3B, right panel). The only significant response in CD4 TCM in the lymph nodes came from treatment with VVΔTK-STCΔNIL-mIL21, which elevated these populations at days 7 and 14 post-treatment (Figure 3B, middle panel), while the combination of VVΔTK-STCΔN1L-mIL21 + α-PD1 elevated CD8+ effector memory T cells (TEM) in the lymph nodes at day 21 significantly compared with all other treatment groups (Figure 3B, left panel). No effect on CD4+ TEM, CD4+ TCM, CD8+ TCM in tumors or CD4+ TEM was noted in lymph node compartments (Figure S2A).

All treatment groups were able to induce M1 macrophage polarization by day 7 in the tumor, reflecting the ability of the virus to remodel the TME to an anti-tumor environment. VVΔTK-STCΔN1L-mIL21 + α-PD1 produced the most significant polarization Figure 3C). A further induction of M1 was noted at day 21 in the tumor, and in the spleen activated M0 macrophages (MHCII+) were detected 21 days post-treatment, which could reflect trafficking of activated macrophages to splenic compartments and activation of the post-oncolytic immune response after the initial phase of immunity. No difference was noted for splenic or lymph node M1 macrophages (Figure S2B).Interestingly, both VVΔTK-STCΔN1L-mIL21 and VVΔTK-STCΔN1L-mIL21 + α-PD1 also increased the activation of DCs in the lymph nodes at day 7, a response that sustained until day 14 (Figure 3C).

VVΔTK-STCΔN1L-mIL21 increased the number of tumor-infiltrated NK cells within the tumor at day 7 after treatment, and the combination with α-PD1 significantly improved this effect. On day 21, NK cells in lymph nodes were elevated by VVΔTK-STCΔN1L-mIL21 + α-PD1 (Figure 3D), but no differences were noted in the spleen compartments (Figure S2C). Of note, effector memory CD8+ T cells within the tumors were significantly increased on days 7 and 21 in the VVΔTK-STCΔN1L-mIL21 and VVΔTK-STCΔN1L-mIL21 + αPD1 groups compared with other groups (Figure 3E).

### VVLΔTK-STCΔN1L-mIL-21 + αPD1 treatment upregulates CD122 in CD8+ tumor-infiltrating T lymphocytes

**Figure 4:**
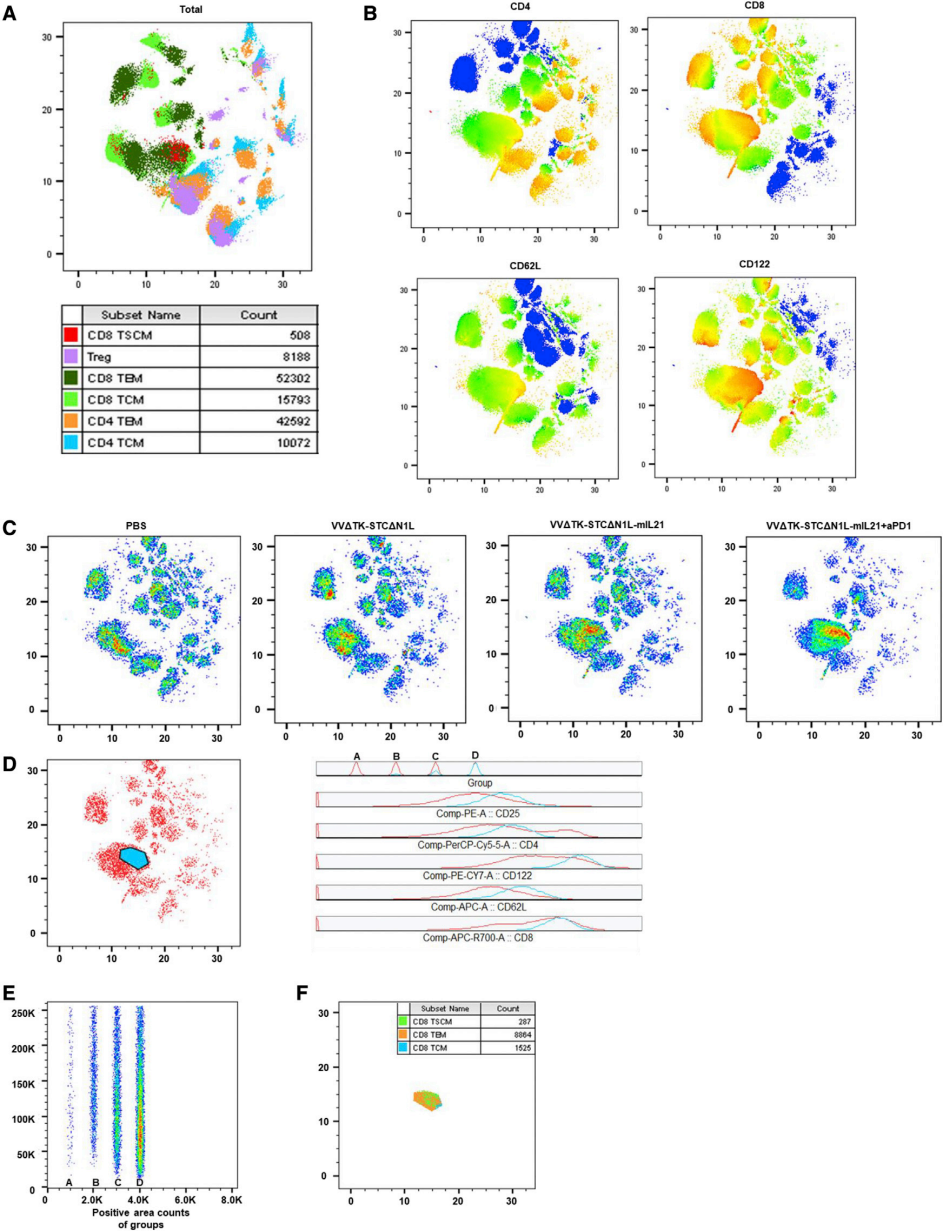
Flow cytometric analysis of T cells in subcutaneous tumor tissue identifies a population of T cells with upregulated CD62L and CD122 expression (A) The dimensionality reduction analysis diagram of subcutaneous tumor tissue cells of all groups at day 7 post-treatment is shown, with the distribution of the main immune cell types. (B) CD4, CD8, CD62L, and CD122 expression heatmap for the combination of all samples. (C) Dimensionality reduction analysis of the combination of all three samples of four treatment groups; cell density is reflected through color (increases from blue to red). (D) The signal expression analysis of the most obvious extra group of cells (blue part). “Group” column was arranged according to the order of treatment groups A (PBS), B (VVΔTK-STCΔN1L), C (VVΔTK-STCΔN1L-mIL21), and D (VVΔTK-STCΔN1L-mIL21 + α-PD1). (E) The count of the cell populations in D of different groups. (F) Immune cell counts (CD3+CD8+CD4-CD62L+CD44-CD122+ CD8TSCM, CD3+CD8+CD4-CD62L-CD44+ CD8TEM, CD3+CD8+CD4-CD62L+CD44+ CD8TCM) of the population marked in D (n = 3–4/group). ∗p < 0.05, ∗∗p < 0.01, ∗∗∗p < 0.001.

To provide an overall and objective view of tumor-infiltrating T cells, here we performed a single-cell flow cytometry analysis. The obtained FACS data were analyzed via conventional gating and Uniform Manifold Approximation and Projection (UMAP) was used to reduce dimensionality of the data and to identify main clusters of immune cells. Analysis of subcutaneous tumor tissues using flow dimensionality reduction analysis at day 7 showed that, in each group that received treatment, an extra group of cells was present (Figures 4A–4C). This was most obvious in the VVΔTK-STCΔN1L-mIL21 + αPD1 group, followed by the VVΔTK-STCΔN1L-mIL21 group, but also present in the VVΔTK-STCΔN1L treatment group. Analysis of this group of cells shows that the cells have phenotypes associated with CD8+ TSCM, TEM, and TCM. The expression of CD122 and CD62L in this group of cells is upregulated (Figures 4D–4F), indicating the presence of antigen-specific activated memory T cells induced by the treatment.

### VVLΔTK-STCΔN1L-mIL-21 activates splenic T cells

**Figure 5:**
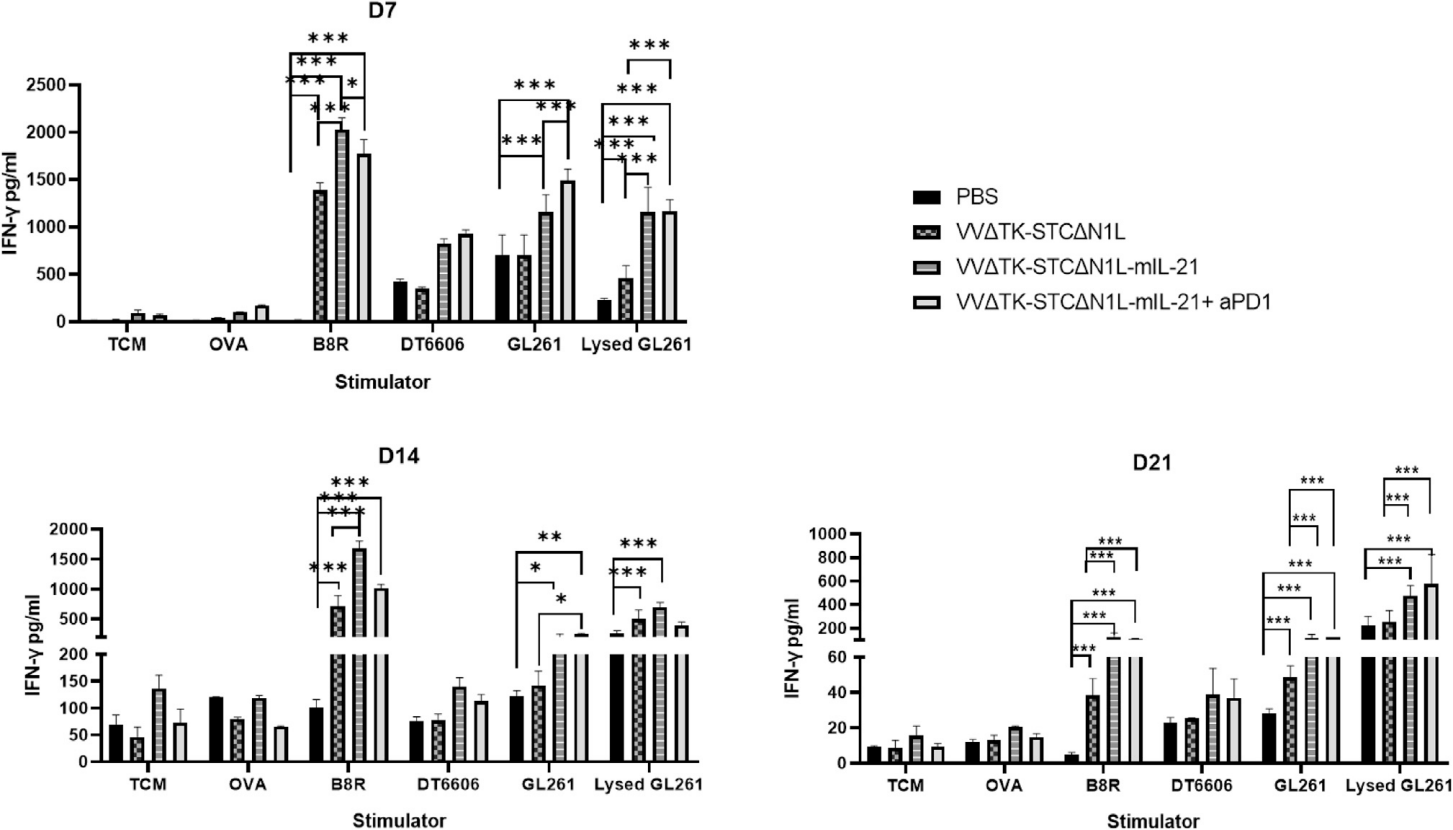
The expression level of IFN-γ in mouse spleen cells after stimulation Splenocytes were harvested on the 7th, 14th, and 21st days after treatment and re-stimulated with the indicated agents. Seventy-two hours after re-stimulation an IFN-γ ELISA was performed. Mean IFN-γ expression ± SEM is shown (n = 3–4/group). A one-way ANOVA was used to determine significant differences between groups ∗p < 0.05, ∗∗p < 0.01, ∗∗∗p < 0.001.

T cell medium (TCM), chicken ovalbumin (OVA), VV peptide B8R, mouse pancreatic cancer cell line DT6606, mouse glioma cell line GL261, and lysed mouse glioma GL261 cells were used for *ex vivo* stimulation of splenocytes harvested on days 7, 14, and 21 post-treatment from treated mice and IFN-γ production determined 72 h later. At each time point, the secretion of IFN-γ was significantly increased after stimulation with the VV peptide B8R, indicating the emergence of an anti-viral response after treatment (Figure 5). Stimulation with GL261 cells and lysed GL261 cells also induced IFN-γ in treatment groups, most significantly in the VVΔTK-STCΔN1L-mIL21 and VVΔTK-STCΔN1L-mIL21 + αPD1 groups (Figure 5). Little IFN-γ was detected after stimulation with unrelated DT6606 cells, indicating that a tumor-specific T cell reactions had been established in response to treatment.

### VVLΔTK-STCΔN1L-mIL-21 and αPD1 co-operate to remodel the TME

**Figure 6:**
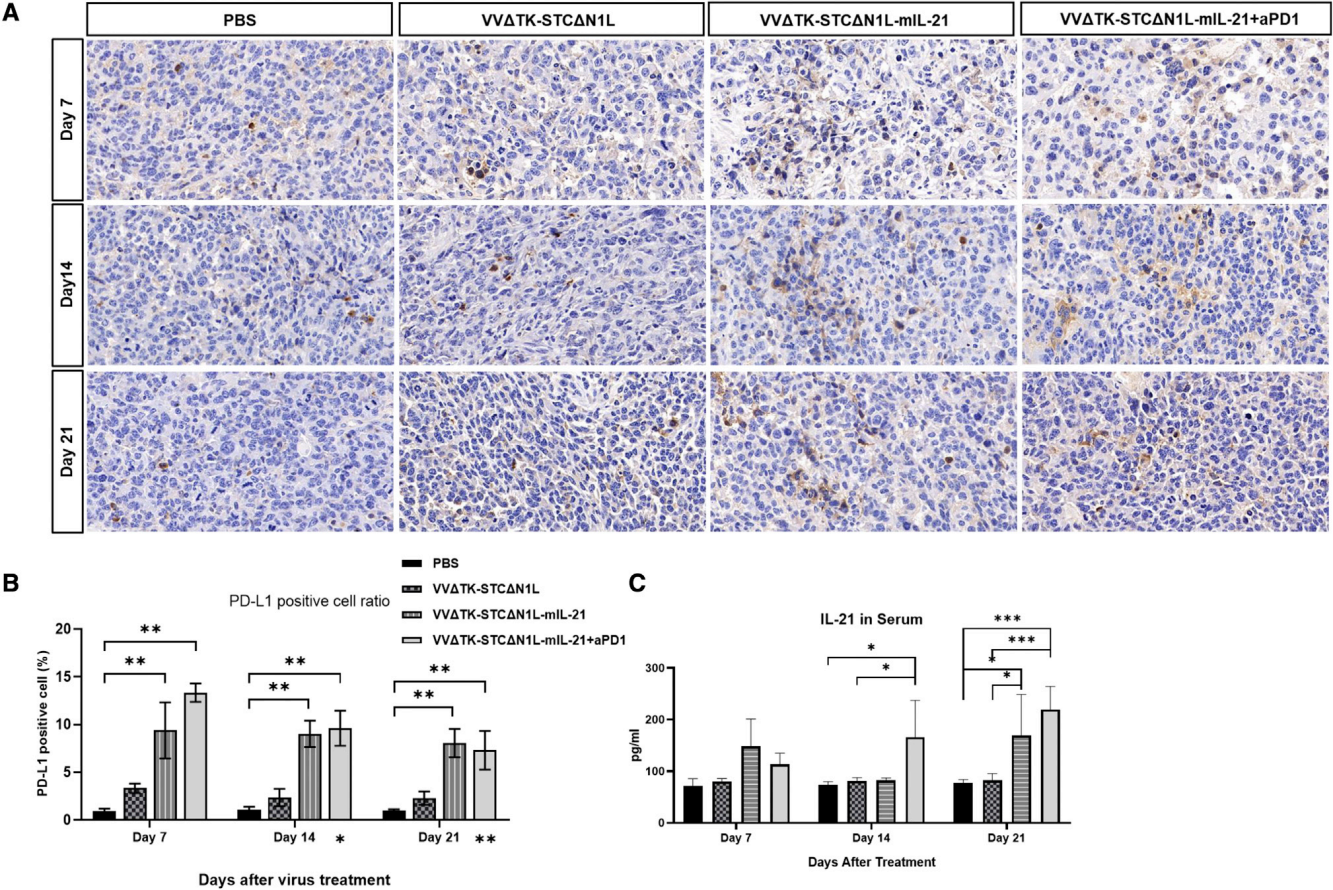
VVΔTK-STCΔN1L-mIL21 and VVΔTK-STCΔN1L-mIL21 + α-PD1 treatment upregulates PD-L1 and mIL-21 expression in murine tumors GL261 tumors were treated in total three times on days 1, 3, and 5 with the indicated virus (±α-PD1) and tumors harvested at indicated time points (days 7, 14, and 21) following the first injection. (A) PD-L1 expression was detected using immunohistochemistry. (B) The proportion of PD-L1-positive cell count to total cell count in each treatment group at three time points. ∗Also, day 14 and day 21 represent the comparison significance of treatment group VVΔTK-STCΔN1L-mIL21 + α-PD1 at days 14 and 21 with day 7, respectively (n = 3/group). Significance was assessed using a one-way ANOVA with Bonferroni post-hoc testing (∗p < 0.05, ∗∗p < 0.01). (C) mIL-21 ELISA assay of the mouse serum at three time points (n = 3/group). Significance was assessed using a one-way ANOVA with Bonferroni post-hoc testing (∗p < 0.05, ∗∗∗p < 0.001).

For each treatment group, the mouse subcutaneous tumors were sectioned at the indicated time points and the expression of PD-L1 was analyzed using immunohistochemistry (IHC) (Figure 6A). Compared with the PBS group and the VVΔTK-STCΔN1L group, the expression of PD-L1 in the tumor was significantly increased after VVΔTK-STCΔN1L-mIL21 and VVΔTK-STCΔN1L-mIL21 + α-PD1 treatment, which gradually decreased as the treatment time passed (Figure 6B). These data indicate that the combination of virus treatment with α-PD1 is critical for treatment success as virus-induced infiltrating T cells are likely to be rapidly deactivated against foreign antigens by virtue of PD1/PD-L1 interactions within the tumor. mIL-21 expression in serum was significantly upregulated after VVΔTK-STCΔN1L-mIL21 treatment on day 14 after treatment and in group VVΔTK-STCΔN1L-mIL21 + α-PD1 on both days 14 and 21, Figure 6C), demonstrating virus-induced expression of the mIL-21 payload.

### VVLΔTK-STCΔN1L-mIL-21 can be delivered intravenously when supported by pharmacological inhibition of PI3Kδ isoform to treat an orthotopic model of glioma

**Figure 7:**
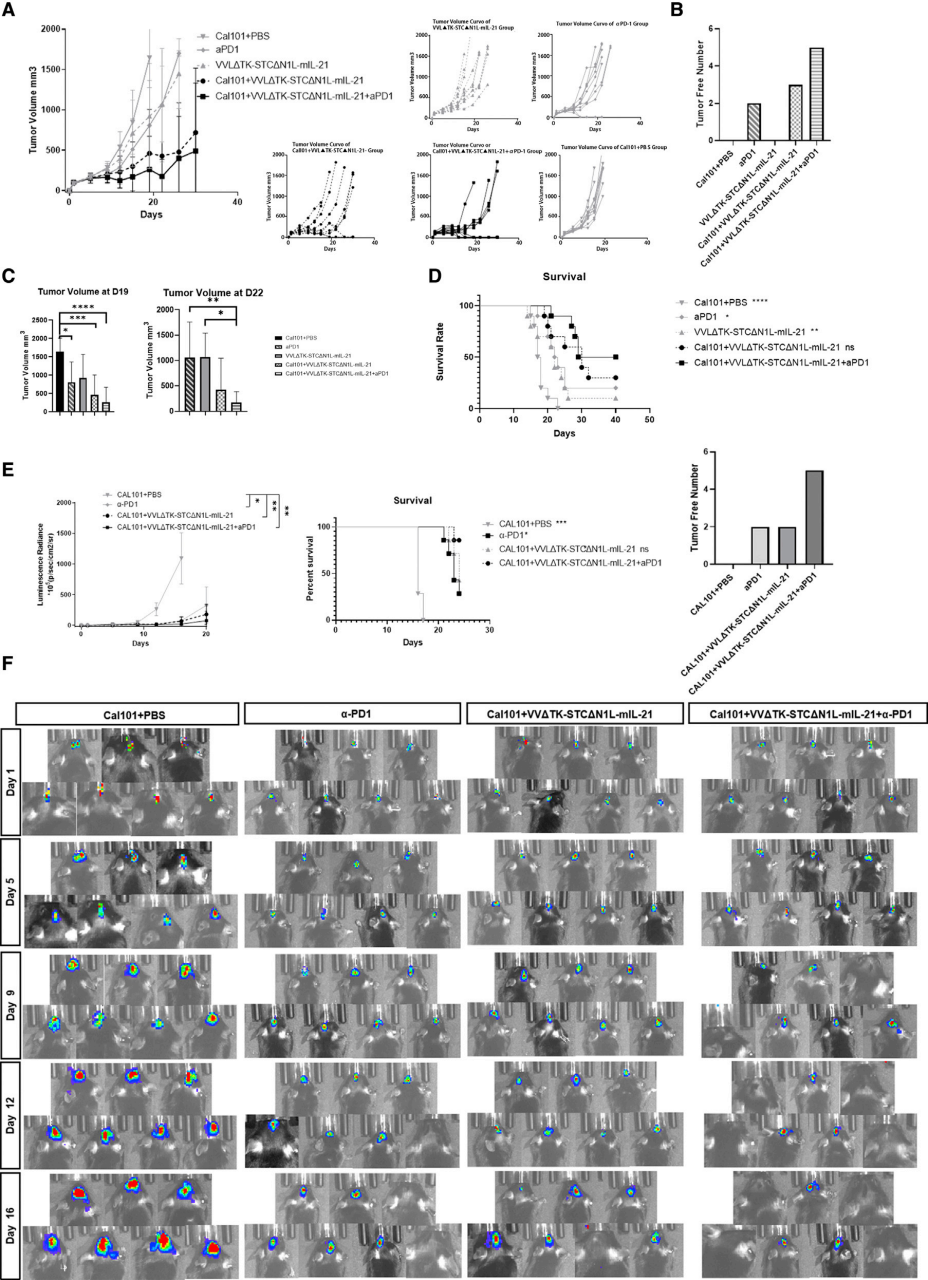
VVLΔTK-STCΔN1L-mIL-21 can be intravenously delivered effectively in GL261 subcutaneous and orthotopic tumor models Mice with established GL261 tumors were treated with the transient PI3Kδ inhibitor CAL101 3 h before intravenous (i.v.) delivery of virus on days 1, 3, 5, 14, 16, and 18. α-PD1 was delivered intraperitoneally (i.p.) on days 2, 4, and 6. One biological repeat was carried out. (A) Tumor growth was monitored and mean tumor size ± SD is presented. Tumor volume curve for each mouse is shown on the right. (B) The number of tumors eliminated after treatment, without recurrence during the observation period, is shown. (C) Tumor volumes at days 19 and 22 after the first treatment, significance was tested using a one-way ANOVA with Bonferroni post-hoc testing. After day 19, many mice in the PBS group were sacrificed due to the tumor reaching maximum limit as defined in the home office license. On day 22, mice in control treatment groups were lost due to the tumor reaching the maximum limit. (D) Kaplan-Meier Survival analysis of mice in each group. Log rank (Mantel-Cox) tests were used to determine significance. The significance in comparison with CAL101+VVLΔTK-STCΔN1L-mIL-21 + α-PD1 group is noted on the legend (n = 10/group). ∗p < 0.05, ∗∗p < 0.01, ∗∗∗p < 0.001, ∗∗∗∗p < 0.0001. (E) GL261-luc cell line mouse orthotopic models were treated using the same strategy described above (one biological repeat was carried out). Tumor luminescence radiance is shown and mean tumor size ± SD is presented (left). Kaplan-Meier survival analysis of mice in each group. Log rank (Mantel-Cox) tests were used to determine significance (middle). The number of orthotopic tumors eliminated is shown on the right panel. The significance in comparison with CAL101+VVLΔTK-STCΔN1L-mIL-21 + α-PD1 group is noted on the legend (n = 7/group). ∗p < 0.05, ∗∗p < 0.01, ∗∗∗p < 0.001, ∗∗∗∗p < 0.0001. (F) *In vivo* bioluminescence imaging of each group at five time points post-treatment. Color changes from blue to red with the increase of radiance intensity.

Considering the intracranial location of GBM, i.v. injection of therapeutics is vital to manage repeated treatments without the risks and side effects associated with craniotomy. Here, we pre-treated animals with a pharmacological inhibitor of PI3Kδ isoform, CAL101, to inhibit macrophage phagocytosis of the virus to increase the systemic delivery of VV as described previously.^21^^,^^24^ We established the GL261-Luc C57BL/6 orthotopic model to recreate the blood-brain barrier (BBB) that may be inhibitory to therapeutic efficacy. The combination of VVLΔTK-STCΔN1L-mIL-21 and α-PD1 was able to suppress tumor growth (Figures 7A, 7C, and 7E), eliminate some of the tumors (Figures 7B and 7E), and significantly increase the survival time of tumor-bearing mice compared with the use of CAL101 alone in both subcutaneous and orthotopic models (Figures 7D and 7E), demonstrating that i.v. delivery is possible, safe, and effective for the treatment of intracranial tumors.

## Discussion

OVs are now recognized as a proven and powerful immunotherapeutic agents, which have the potential to synergize with other immunotherapies and conventional therapies to improve clinical outcomes. VV in particular has many inherent characteristics that make it an ideal anti-tumor agent. By removing the TK and N1L genes (VVLΔTKΔN1L),^24^ tumor specificity of the virus is improved, and potent innate and adaptive immune responses are triggered in the TME. Introducing the mutant form of the virus B5R protein into the TK region, while retaining the unmodified native B5R (VVLΔTKSTCΔN1L), enhances EEV production and therefore virus spreading within and between tumors.^21^

In glioma, the immune microenvironment is strongly suppressive and actively promotes tumor growth. Tumor-associated microglia and macrophages (TAMs) occupy over 30% of the cell population of gliomas.^37^ TAMs, reprogramed as a pro-tumor phenotype, support tumor growth by promoting angiogenesis, proliferation, immune evasion, and therapeutic resistance.^38^, ^39^, ^40^, ^41^, ^42^ Macrophages in glioma demonstrate dynamic phenotypes and M1 or M2 polarization has been noted under different circumstances.^43^, ^44^, ^45^, ^46^ Microglia, abundant in the brain, share the tumor-promoting properties of M2 macrophages and contribute to glioma progression.^47^ Here, we observed that, after treatment with an oncolytic VV armed with the cytokine IL-21, tumor macrophages tend to differentiate more markedly toward an M1 type. This observation has been noted in pancreatic tumors investigated using a similar therapeutic regime and demonstrates that VVLΔTKSTCΔN1L-mIL21 is an effective agent for modifying macrophage phenotypes in immunosuppressive TMEs.^21^ Interestingly, our results demonstrated that, while M1 populations were elevated in glioma tumors at day 7, as expected following infection, we also found an elevation on day 21. This may represent first an acute inflammatory response, followed later by post-oncolytic immunity. In addition, during the acute inflammatory period, the main adaptive immune activation process is the antigen presenting via the lymphatic system. With the progression of the oncolytic effect, more tumor antigens may enter the peripheral blood circulation for capture by splenic monocytes, which can explain the upregulation of activated monocytes in the spleen noted on day 21 post-treatment.

NK cells are strong immune effectors targeting cells lacking MHC class I molecules and have been applied as an anti-tumor agent against gliomas.^48^^,^^49^ In GBM, the low expression of NKG2D results in decreased NK cell activation.^50^ However, studies have illustrated that, even in low numbers, NK cells in glioma can show significant cytotoxicity against tumor cells.^51^ It was noticed in our previous study that IL-21 as well as VVLΔTKΔN1L increased systemic NK cells in multiple tumor models.^21^^,^^23^ In this study, VVLΔTKSTCΔN1L-mIL21 also increased NK cells and the effect was enhanced when combined with α-PD1, demonstrating that multiple tumor-killing mechanisms can be activated using our therapeutic protocol.

After VV enters the tumor, it replicates and kills the tumor cells, resulting in the expression of danger signals that induce strong infiltration and activation of immune cells in the TME to induce anti-tumor immunity. The increase in IFN-γ levels in the TME consequent to infection has previously been shown in other tumors to upregulate the expression of PD-L1 in tumor cells.^52^ Treating cancer with α-PD1 shows clinical benefit in only 20% of patients^53^^,^^54^ and reflects poor activation and infiltration of T cells in many tumors. Indeed, in previous models of GL261 murine glioma, treatment with α-PD1 was unable to alter T cell, NK cell, or macrophage populations.^55^ Thus, treatment with VVLΔTKSTCΔN1L-mIL21 could be expected to enhance the therapeutic landscape of α-PD1 therapies and indeed we demonstrated highly effective anti-tumor responses and improved long-term survival when with α-PD1 therapy was combined with VVLΔTKSTCΔN1L-mIL21 treatment. Indeed, α-PD1 treatment alone was ineffective at inducing long-term survival in our model. The healthy BBB is a diffusion barrier protecting the brain from large compounds and most pathogens. The progression of gliomas depends on neovascularization inducing tortuous, disorganized, and permeable vessels with defective pericyte coverage and an abnormal basement membrane. This remodeling can alter the BBB,^56^ and significant BBB defects caused by hypoxia, necrosis, and immoderate vascular growth is observed in glioma.^56^^,^^57^ However, EGFR upregulation, oxygen deficits, vascularization, and post-surgical damage can effectively enhance the infusion of VV into glioma and increase its tumor selectivity, suggesting that intravenous administration of VV is possible and effective in this disease. Indeed, investigation of treatment efficacy using an orthotopic model, which can be expected to more closely mimic the barriers to effective therapy experienced by glioma patients, demonstrated that VVLΔTKSTCΔN1L-mIL21 was effective (Figure 7E) and safe after i.v. delivery.

After treatment with VVLΔTKSTCΔN1L-mIL21, the number of potent anti-tumor CD4+ and CD8+ TCMs detected in the spleen was significantly increased, an effect that was further enhanced by combining treatment with α-PD1. We also demonstrated that DCs, major antigen-presenting cells critical for anti-tumor immunity, were activated in response to VVLΔTKSTCΔN1L-mIL21 treatment. The effects of VVLΔTKSTCΔN1L-mIL21 on the TME resulted in a pro-immune, anti-tumor environment that provided potent and durable responses against GBM tumors in our murine models. A number of studies have shown that, compared with TEMs and effector T cells, memory T cells, including TSCMs and TCMs, exhibit the most effective durability, resistance, and anti-tumor immunity.^36^ A noteworthy finding was the upregulation of CD122 and CD62L expression on T cells after treatment with VVLΔTKSTCΔN1L-mIL21, indicative of an induction of TSCMs. TSCMs account for only a small proportion of T cells but, in comparison with TCMs, show greater potential to respond to antigen re-stimulation, greater longevity, and a higher capacity for self-renewal. In addition, TSCMs are resistant to cell-cycle arrest and apoptosis after TCR stimulation and have been reported to exert strong anti-tumor activity.^58^ CD8+ TSCMs are able to sustain for more than 25 years and to differentiate into TCM, TEM, and effector T cells.^59^, ^60^, ^61^ After re-stimulation, TSCM cells produce effector CD8+ T cells with high activity and low exhaustion markers, which leads to a powerful anti-tumor response.^62^ One of the major obstacles to efficacy in T-cell based immune therapies is the differentiation and exhaustion of T cells accompanying T cell enrichment.^63^^,^^64^ However, the sustainable tumor regression caused by TSCM has been observed even at low cell numbers,^65^ indicating that TSCM provides a way to avoid T cell exhaustion. This effect is significantly enhanced upon combination with α-PD1 and is essential for the formation of long-term, self-renewable, and stable anti-tumor immunity in the body.

The future for OVT looks positive, and recently OV therapies have been approved for clinical use. A double mutated oncolytic HSV expressing granulocyte macrophage colony-stimulating factor (GM-CSF) has been approved by the FDA and the EMA for the treatment of melanoma.^66^ However, a role for this virus in GBM treatment is precluded as GM-CSF may be a pro-tumor factor through activation of CCL5 in GBM-associated macrophages.^67^ Recently, another HSV vector, G47Δ, was approved in Japan for the treatment of malignant glioma patients.^11^^,^^68^ This virus shows treatment efficacy, but lacks additional payloads that we have shown here can significantly boost CD4+ and CD8+ T cells, especially memory T cell subtypes, which provide long-term robust anti-tumor immunity. In addition, the suitability of VV for i.v. delivery, using our previously described platform,^21^^,^^24^ allows repeated treatment of intracranial tumors in clinical practice without the risks posed by craniotomies.^69^

These results suggest that VVLΔTKSTCΔN1L-mIL21 in combination with α-PD1 could be an extremely effective, systemically deliverable therapeutic option for patients with GBM, expanding the therapeutic options open to these patients and enhancing the therapeutic landscape of clinical available ICI therapies.

## Materials and methods

### Cell lines

The mouse glioma cell line GL-261 and the African green monkey kidney epithelial cell line CV1 were purchased from the American Type Culture Collection (VA, USA). G422 was purchased from the cell bank of the Type Culture Collection Committee of the Chinese Academy of Sciences. GL-261-luc was kindly provided by Professor Yan Dongming from The First Affiliated Hospital of Zhengzhou University, Zhengzhou, China. The cells were cultured in Dulbecco’s modified Eagles medium supplemented with 10% fetal bovine serum and penicillin-streptomycin at 37°C, 5% CO_2_, and saturated humidity. The cells were verified by PCR to be free of mycoplasma contamination.

### Viruses

The virus VVΔTK-STCΔN1L-mIL-21 was described previously.^21^ In brief, this virus is a Lister strain VV with thymidine kinase and N1L gene deletions. A mutant copy of the viral B5R protein (termed STC) is expressed in the TK region under the control of the H5 promoter. The murine IL-21 cytokine is expressed in the N1L region under the control of an H5 promoter.

### VV replication assay

Appropriate cell lines were seeded in triplicate and infected 16 h later with the virus at a MOI of 1 PFU/cell. Cells and supernatant were collected at 24, 48, and 72 h post-infection and titers were determined by measuring the TCID_50_ on indicator CV1 cells. Cytopathic effect was determined by light microscopy 10 days after infection. The Reed-Muench mathematical method was used to calculate the TCID_50_ value for each sample.^70^ Viral burst titers were converted to PFU per cell based on the number of cells present at viral infection. One-way ANOVA followed by Bonferroni post-test was used to assess significance.

### Cell cytotoxicity assay

The cytotoxicity of the viruses in each cell line was assessed in triplicate 6 days after infection with the virus using an MTS non-radioactive cell proliferation assay kit (Promega) according to the manufacturers’ instructions. Cell viability was determined by measuring absorbance at 490 nm using a 96-well plate absorbance reader (Dynex) and a dose-response curve created by non-linear regression allowing the determination of an EC_50_ value (dose required to kill 50% of cells) as described previously.^71^

### *In vivo* experiments

All animal studies carried out were approved by the Animal Welfare and Research Ethics Committee of Zhengzhou University (Zhengzhou, China). To construct the subcutaneous model, 5 × 10^6^ GL261 cells were injected into the right flank of C57BL/6 female mice aged 5–6 weeks. On the tenth day after inoculation, once the tumor volume reached 100 mm^3^, mice were randomly assigned into five groups and treated as described below. Tumor volume was calculated twice a week (V = π × length × width × width/6) until a volume of 1,500 mm^3^ was reached. To establish the orthotopic model, 5 μL, 1 × 10^5^ GL261-luc cells were injected into the right frontal lobe of the mouse within 5 min using a small animal stereotactic frame (SI Instruments). The treatment started on the tenth day after the transplant. The model was monitored twice a week by injecting 0.2 mL 15 mg/mL D-luciferin potassium solution intraperitoneally. The mice were anesthetized using 2.5% isoflurane (R510-22, RWD) at a flow rate of 0.3 L/min. The brain tumor was monitored via bioluminescent imaging using the IVIS Spectrum System (PerkinElmer).

### Treatment of C57Bl/6 mouse subcutaneous GL261 tumor model

PBS, control virus (VVΔTK-STCΔN1L), or therapeutic virus (VVΔTK-STC-ΔN1L-mIL21) were delivered at a dose of 1 × 10^8^ PFU/injection in 100 μL buffer via i.t. injection and multiple injection tracts were followed. Virus was administered on days 1, 3, 5, 14, 16, and 18 after the tumor volume reached 100 mm^3^. α-PD1 (clone G4, kindly donated by professor Shengdian Wang of the Institute of Biophysics, Chinese Academy of Sciences) was delivered at a dose of 200 μg/injection in 200 μL buffer via i.p. injection on days 2, 4, and 6.

### i.v. treatment of the C57Bl/6 mouse subcutaneous and orthotopic GL261/GL261-Luc tumor models

The selective PI3Kδ inhibitor CAL101 was purchased from Selleckchem, re-suspended at 30 mg/mL with 30% PEG 400, 0.5% Tween 80, and 5% propylene glycol, and administered via oral gavage at 10 mg/kg. Virus and α-PD1 were administered as above, but virus was introduced through the caudal vein. Here, we used the CAL101 + VV i.v. injection platform^21^ described previously to examine the efficacy of i.v. treatment using VVLΔTK-STCΔN1L-mIL-21 in combination with α-PD1 on glioma *in vivo*. The mice were treated through caudal vein injection 3 h after CAL101 administration by oral gavage. The animals were treated using 1 × 10^8^ PFU VVLΔTK-STCΔN1L-mIL-21 virus on days 1, 3, 5, 14, 16, and 18, and α-PD1 was applied through i.p. injection on days 2, 4, and 6 after the first treatment.^21^ The volume of the tumor was monitored twice a week.

### Functional studies

Fourteen days after tumor cell inoculation, when the tumor volume grew to 150 ± 20 mm^3^, mice were randomly divided into four groups, each with ten mice. The mice were treated with virus or PBS on days 1, 3, and 5 as above and with α-PD1 on days 2, 4, and 6. On days 7, 14, and 21 post-treatment, organs, peripheral blood, and subcutaneous tumors of mice were collected for follow-up analysis (3–4 mice/group/time point).

### IFN-γ release assay of spleen cells

Harvested spleens were flushed through 70 μm BD Falcon cell strainers with complete TCM medium (RPMI medium 1640; Sigma-Aldrich), 10% FCS, 1% streptomycin/penicillin, 1% sodium pyruvate, 1% non-essential amino acids (Gibco), 100 IU/μL rhIL-2 (Peprotech), and 50 μM (β-mercaptoethanol). Red blood cells were lysed using RBC lysis buffer (Sigma-Aldrich) and re-suspended in TCM. Cells (100 μL 5 × 10^5^) were added into each well of 96-well plate and co-cultured with 100 μL 10 μg OVA peptide (SIINFEKL), volume 100 μL, final concentration 10 μg B8R peptide per well (TSYKFESV), whole DT6606 cells, GL261 cells, or MMC-treated GL261 cells in 100 μL TCM. After 72 h, the 96-well plate was centrifuged at 1,600 rpm (approximately 273 g) for 5 min and the supernatant was used in an ELISA (Invitrogen; 88-7314-88; Thermo Fisher Scientific) to detect the concentration of IFN-γ according to the manufacturers’ instructions.

### Flow cytometry analysis of immune cell populations

Each sample was stained according to the manufacturers’ protocol (all antibodies were from BioLegend; Dakewe Biotech). Tube A (CD4+ and CD8+ populations): FITC anti-mouse CD3 (0.5 mg/mL; cat. no. 100204), PE anti-mouse CD25 (0.2 mg/mL; cat. no. 101904), PerCP/Cyanine5.5 anti-mouse CD4 (0.2 mg/mL; cat. no. 100434), APC anti-mouse CD62L (0.2 mg/mL; cat. no. 104412), Brilliant Violet 605 anti-mouse/human CD44 (0.2 mg/mL; cat. no. 103047), Alexa Fluor 700 anti-mouse CD8a (0.5 mg/mL; cat. no. 100730), PE/Cyanine7 anti-mouse CD122 (IL-2Rβ) (0.2 mg/mL; cat. no. 123216). Tube B (Macrophage, DC populations): PE anti-mouse CD86 (0.2 mg/mL; cat. no. 159204), APC anti-mouse F4/80 (0.2 mg/mL; cat. no. 123116), FITC anti-mouse/human CD11b (0.5 mg/mL; cat. no. 101206), PerCP/Cyanine5.5 anti-mouse CD11c (0.2 mg/mL; cat. no. 117328), Alexa Fluor 700 anti-mouse I-A/I-E (0.5 mg/mL; cat. no. 107622), PE/Cyanine7 anti-mouse CD206 (MMR) (0.2 mg/mL; cat. no. 141720). Tube C (NK populations): PE anti-mouse NK-1.1(0.2 mg/mL; cat. no. 108708). Tubes B and C were blocked with 10% goat serum (Beyotime; Bio SCI Bio) for 30 min, centrifuged at 3,000 rpm (approximately 845 × g) for 5 min and the supernatant removed. After antibodies were added, tubes were incubated on ice for 30 min in the dark before the samples were centrifuged at 3,000 rpm (approximately 845 × g) for 5 min. After centrifugation, the supernatant was discarded, the volume adjusted to 1 mL with PBS, and the cells were re-suspended by pipetting before analysis.

### Pathological paraffin section and immunohistochemistry

Appropriately harvested samples were immediately fixed in 4% w/v formalin (diluted with PBS) for 48 h. The samples were embedded in paraffin, then following a standard procedure of immunohistochemistry (IHC) staining for detection of PDL-expression using a Rat-anti-mouse PD-L1 antibody (BioLegend; 124302).^71^ In brief, 4 μm paraffin sections were made and blocked with 3% BSA and incubated with the 100× diluted PD-L1 antibody, washed, and incubated with anti-rat antibody conjugated with HRP, then stained with DAB chromogen solution and followed with hematoxylin counterstain. Four 40× areas were selected for each sample analyzed by ImageJ v.2.1.0. The number of cell nucleus was counted to represent cell number. Each positive area surrounding the nucleus was defined as one positive cell. The mean of the ratio between positive cell and cell number in the four areas selected stands for the PD-L1 positive percentage value of the sample.

### mIL-21 and IFN-γ ELISA

ELISAs were carried out using an Invitrogen Mouse IL-21 Uncoated ELISA Kit (cat. no. 88-8210-22) and a Mouse IFN-γ ELISA Kit (cat. no. 88-7314-88) according to the manufacturers’ instructions.

### Statistical analysis

Tumor cell flow cytometry data were analyzed by FlowJo_v10.7.1. For UMAP (using UMAP plugin v.3.1^72^) analysis, data files were passed through a process, including cleanup for viability, cell aggregates, and instrument acquisition anomalies, using a combination of manual gating and the Flow AI v.2.2 plugin (FlowJo Exchange).^73^ Files were down-sampled (Down Sample v.3.3 plugin^72^) to a fixed number of single cells at 10,000 events per sample. Down-sampled events were concatenated into a single file and the UMAP algorithm was applied using all antibodies in the panel as parameter input values. All processes above were carried out with publicly available Flow Jo Plugins. Statistical analyses were performed using GraphPad Prism 8.0. The results were represented as mean ± SD. Comparison between groups was performed using t test, one-way ANOVA, two-way ANOVA, or Kaplan-Meier survival analysis as described in the figure legend. p < 0.05 was considered significant.

## Supplemental information

Document S1. Figures S1 and S2

Document S2. Article plus supplemental information
