# Supplementary material for: An effective therapeutic regime for treatment of glioma using oncolytic vaccinia virus expressing IL-21 in combination with immune checkpoint inhibition
Source: Mol Ther Oncolytics. 2022 Jun 6;26:105–19. doi: 10.1016/j.omto.2022.05.008 (PMC9233193; doi:10.1016/j.omto.2022.05.008)
Supplement: Document S1. Figures S1 and S2 [file mmc1.pdf]

OMTO, Volume *26*

Supplemental information

An effective therapeutic regime for treatment of glioma using oncolytic vaccinia virus expressing IL-21 in combination with immune checkpoint inhibition

Yijie Sun, Zhe Zhang, Chenglin Zhang, Na Zhang, Pengju Wang, Yongchao Chu, Louisa S. Chard Dunmall, Nicholas R. Lemoine, and Yaohe Wang

A

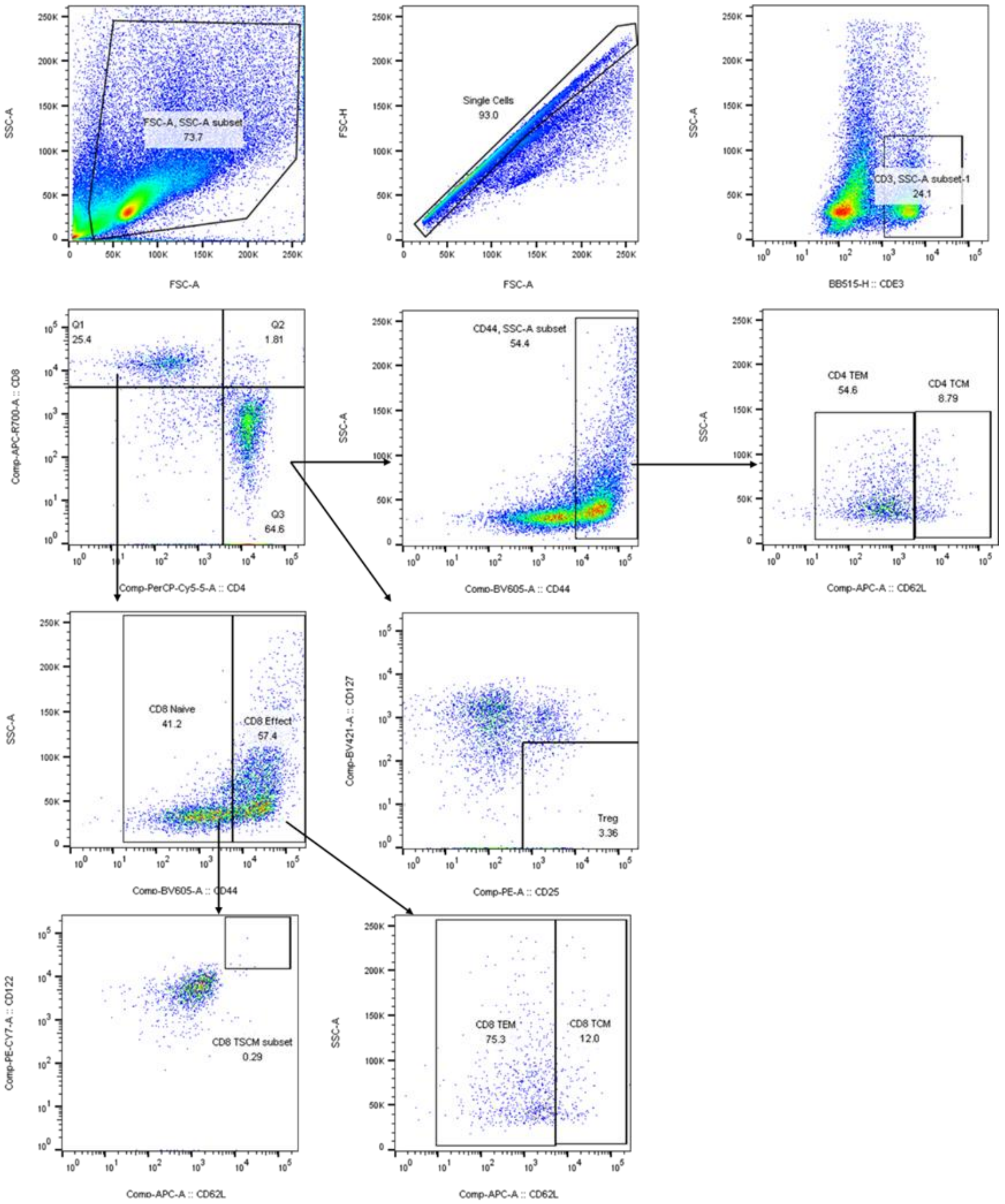

FSC-A, SSC-A subset
73.7
SSC-A
FSC-A
Single Cells
93.0
FSC-H
CD3, SSC-A subset-1
24.1
BB515-H :: CDE3
Q1
25.4
Q2
1.81
Q3
64.6
Comp-APC-R700-A :: CD8
Comp-PerCP-Cy5-5-A :: CD4
CD44, SSC-A subset
54.4
Comp-BV605-A :: CD44
CD4 TEM
54.6
CD4 TCM
8.79
Comp-APC-A :: CD62L
CD8 Naive
41.2
CD8 Effect
57.4
Comp-BV421-A :: CD127
Comp-PE-A :: CD25
Treg
3.36
Comp-PE-CY7-A :: CD122
CD8 TSCM subset
0.29
CD8 TEM
75.3
CD8 TCM
12.0

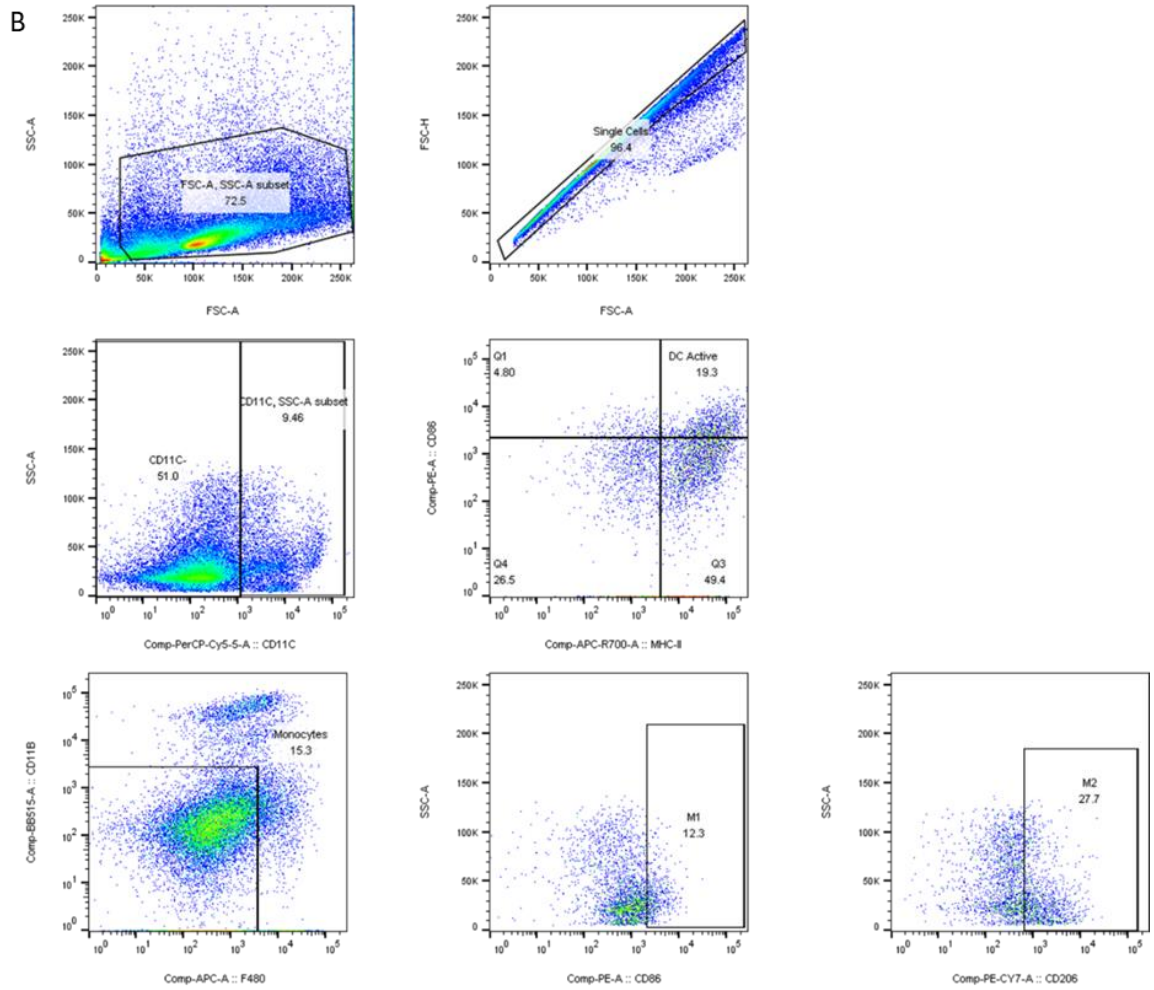

Figure S1 Cell flow cytometry gating. Murine glioma Gl261 subcutaneous tumors were established and treated with PBS, VVΔTK-STCΔN1L, VVΔTK-STCΔN1L-mIL21 or VVΔTK-STCΔN1L-mIL21+ α -PD1 on days 1, 3, 5 as described in the methods. On days 7, 14 and 21 post-treatment, different organs, peripheral blood and subcutaneous tumors of mice were collected for FACS analysis (3 mice/group/timepoint). A: T cell flow cytometry gating. B: Dendritic cell (DC) macrophage (MO) circle gate path diagram.

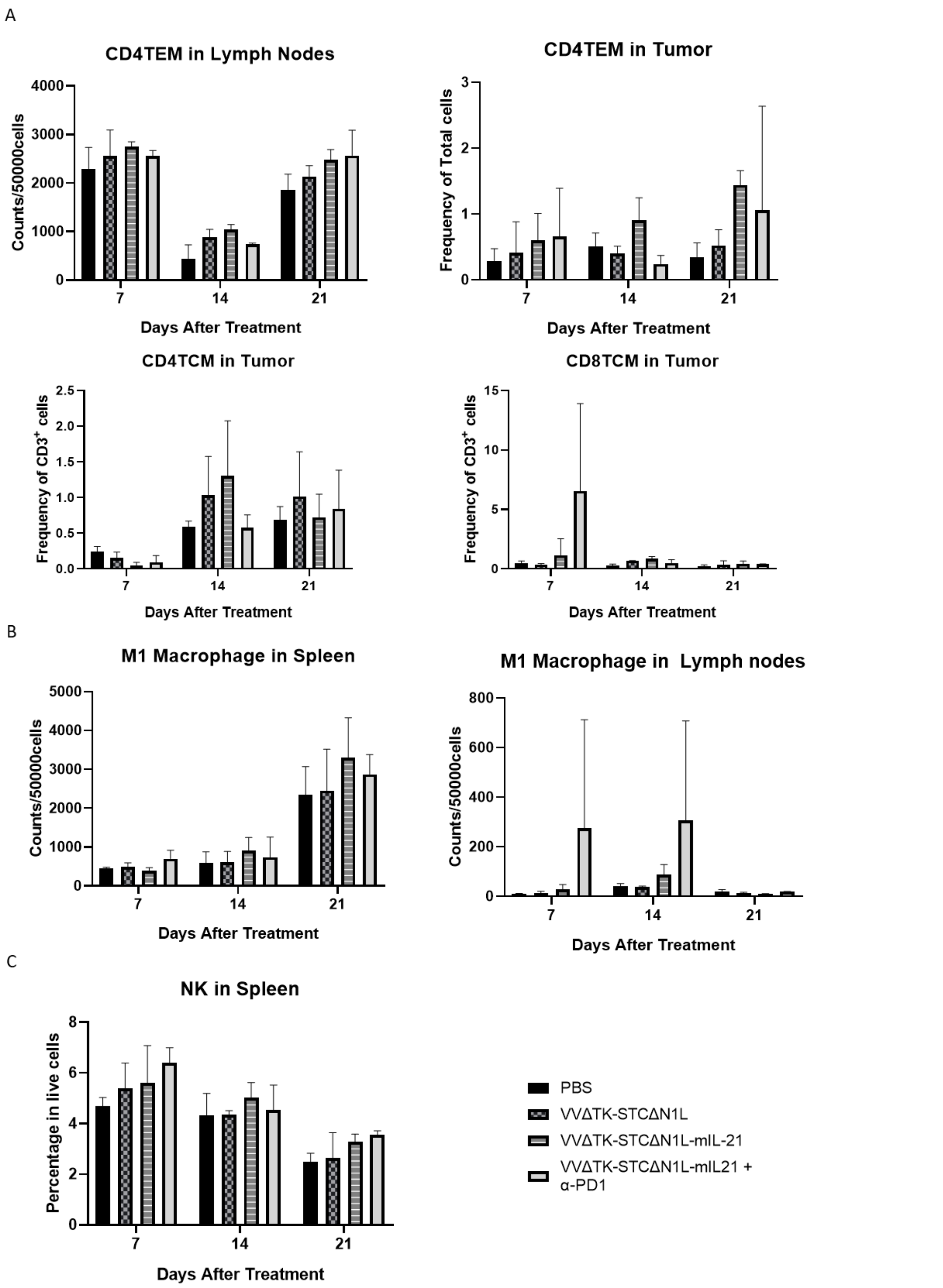

Figure S2 Immune cells subtypes of treatment groups. (A) CD4 TEM cells in the lymph nodes and tumors of mice. (N=3-4/group. Frequency (%) of total cells or CD3+ cells was used to evaluate CD4⁺TEM, CD4⁺TCM and CD8⁺TCM because of the low amount of the cell subset infiltrated in the tumor. (B)M1 macrophages quantified in the spleens and lymph nodules at the indicated timepoints after treatment. (C) NK cells were quantified in the spleen at the indicated timepoints after treatment. Significance was analysed using a two-way ANOVA, * P＜0.05; ** P＜ 0.01,***P<0.001)
